# Impact of Neoadjuvant Chemotherapy on the Administration of Concurrent Chemoradiation for Locally Advanced Nasopharyngeal Carcinoma

**DOI:** 10.7759/cureus.2971

**Published:** 2018-07-12

**Authors:** Benjamin Maas, Cheryl Ho, Sarah Hamilton, Doug Leedy, Eric Berthelet

**Affiliations:** 1 Radiation Oncology, British Columbia Cancer, Vancouver Cancer Centre, Vancouver, CAN; 2 Medical Oncology, British Columbia Cancer, Vancouver Cancer Centre, Vancouver, CAN; 3 Medicine, University of British Columbia, Vancouver, CAN

**Keywords:** nasopharyngeal carcinoma, neoadjuvant chemotherapy, concurrent chemotherapy

## Abstract

Objectives

The standard of care for locally advanced nasopharyngeal carcinoma (NPC) is concurrent cisplatin chemoradiotherapy. Neoadjuvant chemotherapy can be administered to downsize tumors before concurrent treatment to optimize radiation volumes. Our hypothesis was that the use of cisplatin in the neoadjuvant phase could limit the amount of cisplatin that patients could tolerate in the concurrent phase of treatment.

Methods

This is a retrospective analysis of Canadian NPC patients who received neoadjuvant chemotherapy with the intention to downsize locally advanced tumors prior to concurrent cisplatin plus radiation. Baseline demographic and treatment data were obtained from institutional databases and chart review; all data were analyzed with SPSS (SPSS Inc. Released 2005. SPSS for Windows, Version 14.0. Chicago: SPSS Inc.) Overall survival (OS), disease-specific survival (DSS), and local/regional relapse-free survival (LRRFS) were analyzed using Kaplan-Meier survival functions. Univariate and multivariate models were used to determine factors associated with the total dose of concurrent chemotherapy.

Results

Forty-six patients were identified as receiving neoadjuvant chemotherapy before concurrent chemoradiotherapy. In the univariate and multivariate analyses of patients who received concurrent chemotherapy, receiving over 200 mg/m^2^ concurrent cisplatin with radiation was associated with a higher neoadjuvant dose of chemotherapy received. The median follow-up time was 2.6 years (range, 0.17 years to 10.6 years). At three years, the OS was 83%, DSS was 86%, and LRRFS was 74%.

Conclusions

NPC patients have been treated with neoadjuvant chemotherapy at this center with favorable outcomes. Most patients could tolerate concurrent chemotherapy after radiotherapy. Receiving higher doses of concurrent chemotherapy was associated with also having higher doses of neoadjuvant cisplatin. This suggests that neoadjuvant cisplatin is not a limiting factor in the delivery of full-dose concurrent chemotherapy.

## Introduction

Radiotherapy is the mainstay of treatment for nasopharyngeal carcinoma (NPC) [1-2]. Doses of 70 Gy directed to the primary tumor and involved lymph nodes have been recognized as a curative treatment protocol. In the locally advanced setting, concurrent chemoradiotherapy is the standard of care based on Phase III trials conducted in both North America and Asia that demonstrated a significant survival benefit [3-4]. The most common radiosensitizing agent is cisplatin delivered at 40 mg/m^2^ weekly or 100 mg/m^2^ every three weeks. The roles of neoadjuvant and adjuvant chemotherapy remain controversial, with conflicting results throughout the literature [1,5-9].

Neoadjuvant chemotherapy has been used for locally advanced presentations of NPC. The proposed benefits of neoadjuvant chemotherapy in this setting are to downsize the tumor prior to radiation and to treat microscopic metastatic disease. In bulky Stage III and Stage IV-A and IV-B tumors, encompassing the gross tumor volume (GTV) with a curative dose of 70 Gy may prove challenging due to the proximity of dose-limiting structures, such as the spinal cord, brain stem, brain, and optic structures. Downsizing from neoadjuvant chemotherapy may help shrink the tumor away from dose-limiting critical structures and, therefore, facilitate radiation delivery and reduce the risk of significant morbidity or even mortality.

There are cumulative toxicities associated with higher total doses of platinum-based chemotherapy, including ototoxicity, nephrotoxicity, and neuropathy. It is unclear if increasing the total chemotherapy dose by adding a neoadjuvant phase limits a patient’s ability to complete the curative-intent concurrent treatment phase. Because the efficacy of concurrent chemoradiotherapy for NPC has been well-demonstrated, it is important to assess whether neoadjuvant platinum-based chemotherapy lowers a patient’s ability to receive concurrent chemotherapy with radiation.

At the British Columbia Cancer Agency (BCCA), selected patients with bulky NPC primary tumors at diagnosis have been treated with the neoadjuvant chemotherapy phase prior to definitive concurrent chemoradiation. We conducted a retrospective analysis to identify all such patients and report on the clinical outcomes. The primary objectives were to present a Canadian experience with neoadjuvant chemotherapy and to characterize the subsequent ability to complete the intended and prescribed concurrent chemotherapy during radiation. The secondary objectives were to report the disease recurrence and survival outcomes for the patients treated.

## Materials and methods

A retrospective review was conducted of patients diagnosed with NPC treated at the BCCA from January 1, 2000, to December 1, 2013, who received chemotherapy prior to planned curative intent concurrent chemoradiation treatment. The BCCA has six regional centers and provides comprehensive oncologic care to a population of 4.5 million people, which includes chemotherapeutic drugs and all radiation therapy. This large population and single radiation provider allow for prospective data capture on treatments and outcomes for a population-based assessment of outcomes.

Demographic data obtained from patient medical records included age, sex, ethnicity, date of birth, smoking status, and Eastern Cooperative Oncology Group (ECOG) performance status. Disease characteristics obtained included tumor-node-metastasis (TNM) stage, American Joint Committee on Cancer 2009 stage, and International Classification of Diseases diagnosis codes.

Neoadjuvant chemotherapy was defined as any chemotherapy regimen received prior to concurrent chemoradiotherapy. All neoadjuvant protocols were platinum-based. Patients were selected for neoadjuvant chemotherapy if they had bulky T3/T4 disease invading or abutting the brain, optic structures, brain stem, or spinal cord to cytoreduce the bulk of disease prior to the delivery of radiotherapy. All patients were assessed by a medical oncologist for suitability for neoadjuvant chemotherapy and were ECOG 0-2 with adequate hematologic, hepatic, and renal function. Standard concurrent chemotherapy regimens utilized were cisplatin 40 mg/m^2^ weekly for seven cycles or cisplatin 100 mg/m^2^ every three weeks for three cycles. Treatment data were compiled, including start date, end date, chemotherapy agents (neoadjuvant and concurrent), the total dose of cisplatin in mg/m^2^, and the number of cycles. Radiotherapy is most commonly prescribed as 70 Gy in 35 daily fractions five days per week, with some physician variation in dose and fractionation.

A cutoff of ≤ 200 mg/m^2^ or > 200 mg/m^2^ of cisplatin received during the concurrent phase was used to stratify the cohort. This was selected as a marker for completing at least two thrice-weekly 100 mg/m^2^ cycles or five weekly 40 mg/m^2^ cycles of once weekly concurrent cisplatin. Univariate and multivariate models were used to determine factors associated with receiving < 200 mg/m^2^ of concurrent chemotherapy.

Secondary endpoints included overall survival (OS), disease-specific survival (DSS), and local/regional relapse-free survival (LRRFS). OS was defined as the duration from diagnosis to the date of death from any cause or censored at last date of follow-up. DSS was defined as the time from diagnosis to death from NPC. Local recurrence was defined as any recurrence in the nasopharynx after a treatment response as identified by clinical exam, imaging, or biopsy. Regional recurrence was defined as any recurrence in regional nodes. LRRFS was defined as time to a local or regional recurrence.

Categorical variables were compared using Fisher’s exact test, and continuous variables were analyzed using the Mann-Whitney test. Survival was analyzed with Kaplan-Meier survival functions. The three-year survival endpoints were extracted. Patients with longer than three years of follow-up were censored at three years for this subanalysis. Statistical analysis was conducted using SPSS (SPSS Inc. Released 2005. SPSS for Windows, Version 14.0. Chicago: SPSS Inc.).

The study was reviewed and approved by the BCCA Research Ethics Board.

## Results

From January 2000 to December 2013, 428 cases of NPC were identified at the BCCA. Of these, 46 patients fit the inclusion criteria of receiving neoadjuvant chemotherapy prior to planned concurrent chemoradiotherapy. The baseline characteristics of the 46 patients, including demographic and tumor staging information, are found in Table 1. The majority of patients were male, of Asian ethnicity, and had T3 or T4 tumors with involved nodes.

**Table 1:** Patient and tumor characteristics Abbreviations: ECOG, Eastern Cooperative Oncology Group.

_Age_	_Mean: 47 years _	
	_Range: 18 – 75 years_	
_Gender_	_Actual _	_Percent_
_Female_	_9_	_20%_
_Male_	_37_	_80%_
_Ethnicity_		
_Caucasian_	_12_	_26%_
_Asian_	_34_	_74%_
_ECOG Performance Status_		
_0_	_17_	_37%_
_1_	_23_	_50%_
_2_	_6_	_13%_
_Tumor Stage_		
_2_	_3_	_7%_
_3_	_10_	_22%_
_4_	_33_	_72%_
_Nodal Stage_		
_0_	_9_	_20%_
_1_	_14_	_30%_
_2_	_20_	_43%_
_3_	_3_	_7%_
_Metastases_		
_0_	_46_	_100%_
_Stage_		
_III_	_12_	_26%_
_IV_	_34_	_73%_
_Smoking Status_		
_Current_	_8_	_17%_
_Former_	_12_	_26%_
_Never_	_22_	_48%_
_Unknown_	_4_	^9%^

Treatment details are outlined in Table 2. The most common neoadjuvant chemotherapy combination was cisplatin and gemcitabine. The median dose of neoadjuvant cisplatin was 160 mg/m^2^ (range, 24 mg/m^2^ to 325 mg/m_2_). Forty-three patients were scheduled to receive cisplatin 40 mg/m^2^ weekly for seven cycles, and three patients were scheduled to receive cisplatin 100 mg/m^2^ every three weeks for three cycles. Of the 46 patients, 52% received all planned concurrent cisplatin. The median dose of concurrent cisplatin was 195 mg/m^2^ (range, 67 mg/m^2^ to 397 mg/m^2^). Twenty-one of 46 patients received ≤ 200 mg/m^_2_^ concurrent cisplatin, including four patients who did not receive any of the concurrent phase planned cisplatin. Some patients who received less than 70 Gy were due to the proximity of the tumor to organs of interest (OARs); no patient stopped radiation treatment early due to toxicity. One patient declined radiation due to fear of potential side effects.

**Table 2:** Neoadjuvant chemotherapy regimen, dose of concurrent chemotherapy, and radiation dose fractionation schedule Abbreviations: RT, radiotherapy.

Neoadjuvant Chemotherapy Agents	Number of Patients	Percent of Patients
Gemcitabine & Cisplatin	40	87%
Etoposide & Cisplatin	3	6.5%
Fluorouracil & Cisplatin	3	6.5%
Concurrent Chemotherapy (Planned Treatment)		
Cisplatin 40 mg/m^2^ weekly x 7	43	94%
Cisplatin 100 mg/m^2^ every 3 weeks x 3	3	6%
Number of patients who received all planned Cisplatin	24	52%
Cisplatin Dose Stratified		
≤ 200 mg/m^2^ (Range: 0-200mg/m^2^)	21	46%
> 200 mg/m^2 ^(Range: 201-320mg/m^2^)	25	54%
Radiation Dose and Fractionation		
70 Gy / 35#	33	72%
70 Gy / 36#	1	2%
70 Gy / 33#	1	2%
66 Gy / 33#	4	9%
60 Gy / 30#	3	7%
60 Gy / 25#	2	4%
56 Gy / 28#	1	2%
Declined RT	1	2%

Of the 46 patients who received neoadjuvant chemotherapy, 45 went on to receive radiation therapy. One patient refused any radiation treatment after the neoadjuvant treatment phase. All were given radical radiotherapy courses, but there was some variability in the dose and fractionation based on provider choice and patient-specific factors. Radiation doses ranged from 56 Gy to 70 Gy in 25 to 36 daily fractions (Table 2). The most common dose fractionation was 70 Gy in 35 fractions for 33 patients (72%).

Potential factors associated with completing > 200mg/m^2^ concurrent phase cisplatin were analyzed in univariate and multivariate models presented in Table 3. Receiving a higher dose of neoadjuvant chemotherapy was significantly associated with completing > 200 mg/m^2^ concurrent cisplatin in the univariate analysis. Males also trended towards higher concurrent chemotherapy strata with a p-value of 0.059. Stage, age, ECOG performance status, and ethnicity were not predictive of completing concurrent chemotherapy.

**Table 3:** Univariate and multivariate by dose stratification Abbreviations: ECOG, Eastern Cooperative Oncology Group; NA, not applicable.

	Univariate			Multivariate	
	≤ 200 mg/m^2^	> 200 mg/m^2^	p-value	Odds Ratio	p-value
Sex					
Female	6 (35%)	2 (8%)	0.045	4.9	0.027
Male	11 (65%)	23 (92%)			
Ethnicity					
Caucasian	5 (30%)	4(16%)	0.45	NA	NA
Asian	12 (71%)	21 (84%)			
Stage					
III	5 (29%)	7 (28%)	1		
IV	12 (71%)	18 (72%)			
ECOG Performance Status					
0	17 (100%)	21 (84%)	0.13	NA	NA
1 or 2	0 (0%)	4 (16%)			
_Smoking Status_					
_Current/former_	_12 (50%)_	_9 (50%)_	_0.7_	_NA_	
_Never_	_9 (38%)_	_8 (44%)_			
_Unknown_	_3 (13%)_	_1 (6%)_			
Mann-Whitney Test	Mean Rank	Mean Rank			
Age	23.8	19.9	0.32	NA	NA
Neaodjuvant cisplatin (mg/m^2^)	15.6	25.5	0.01	7.2	0.007

The multivariate model included gender and the dose of neoadjuvant chemotherapy from the univariate models. A linear regression analysis of the continuous dose of cisplatin received neoadjuvantly and the dose of cisplatin received concurrently found a positive correlation; patients who were able to receive higher doses of cisplatin in the neoadjuvant setting also received higher doses of cisplatin in the concurrent setting.

**Figure 1:**
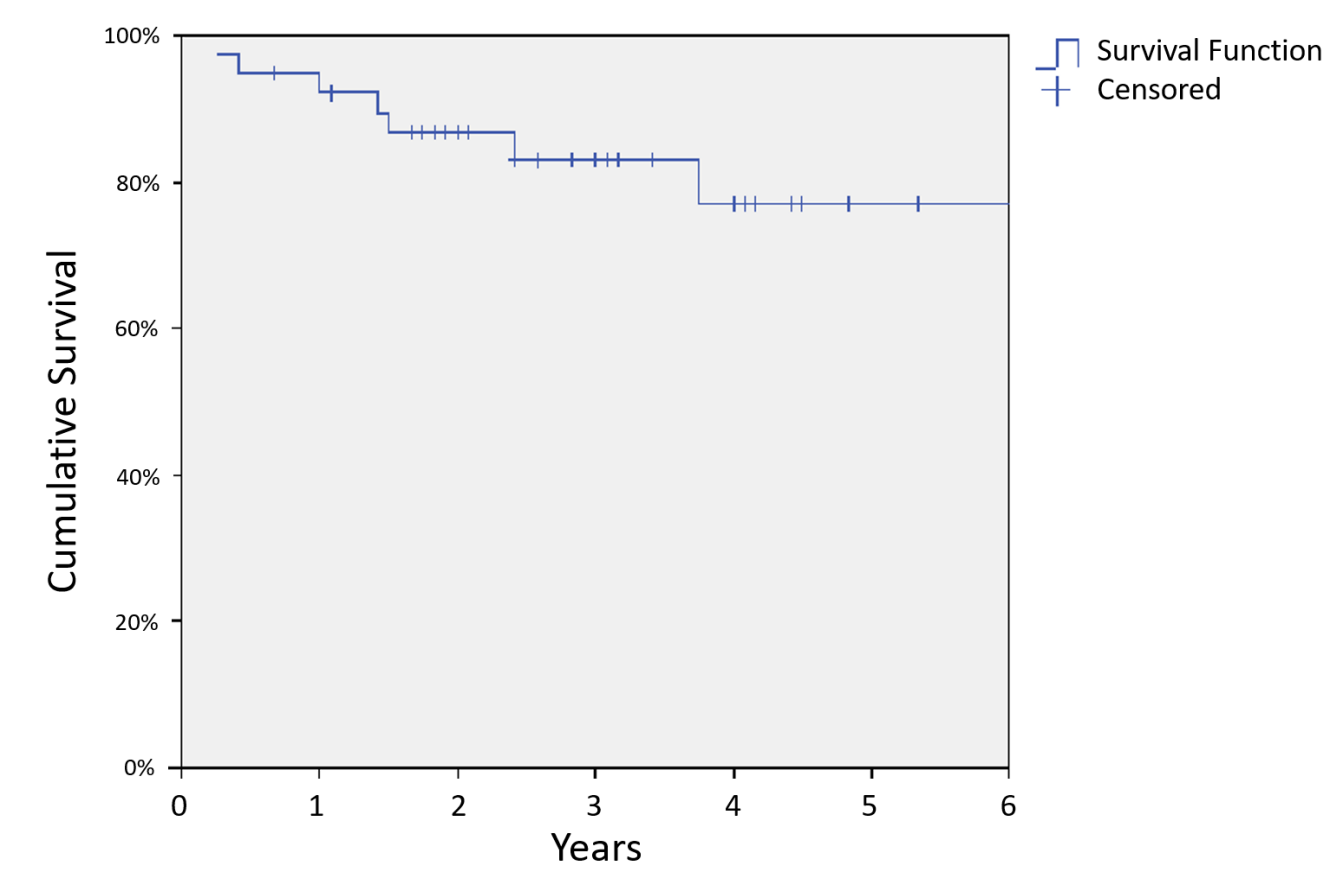
Overall survival

**Figure 2:**
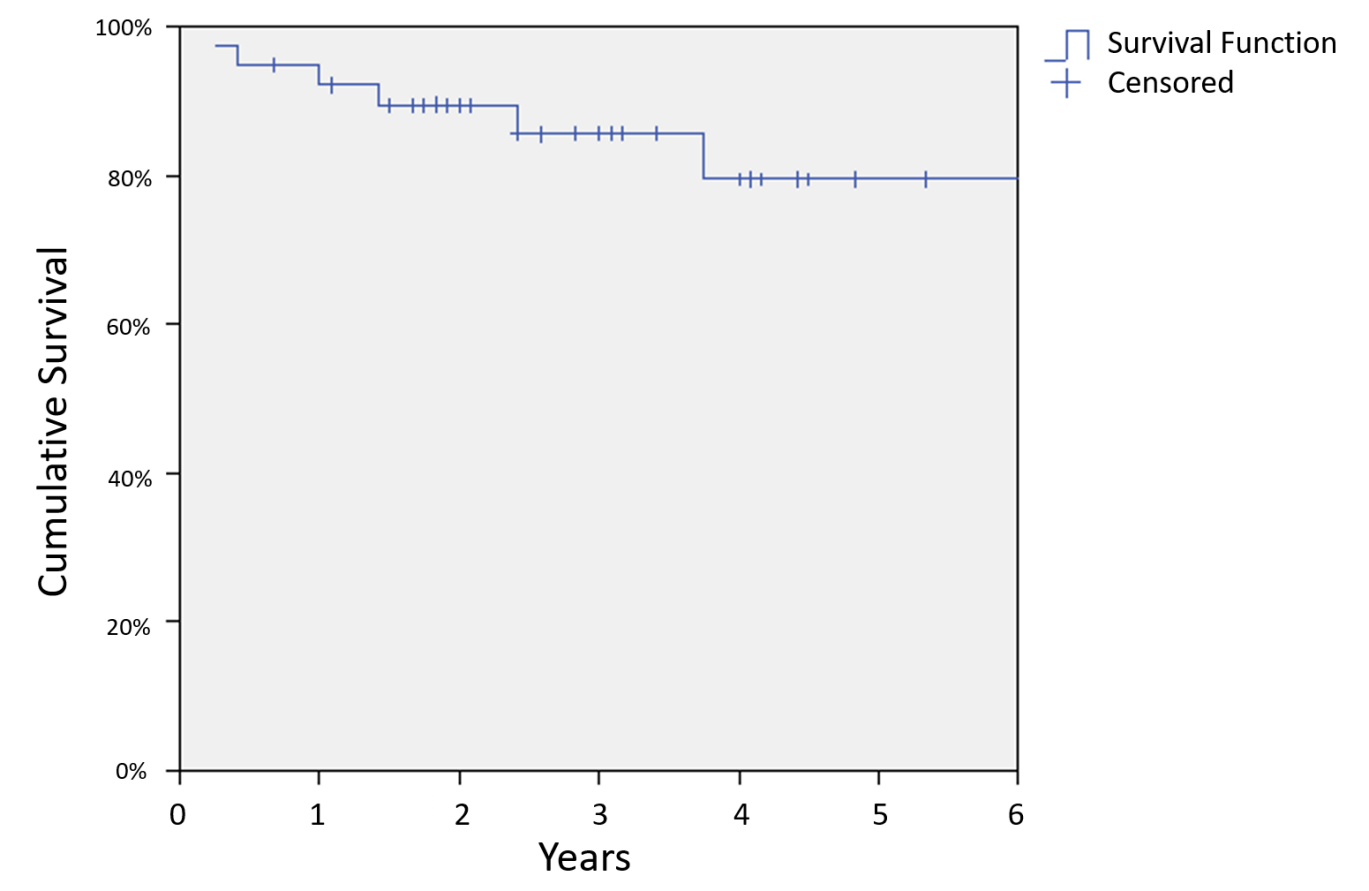
Disease-specific survival

**Figure 3:**
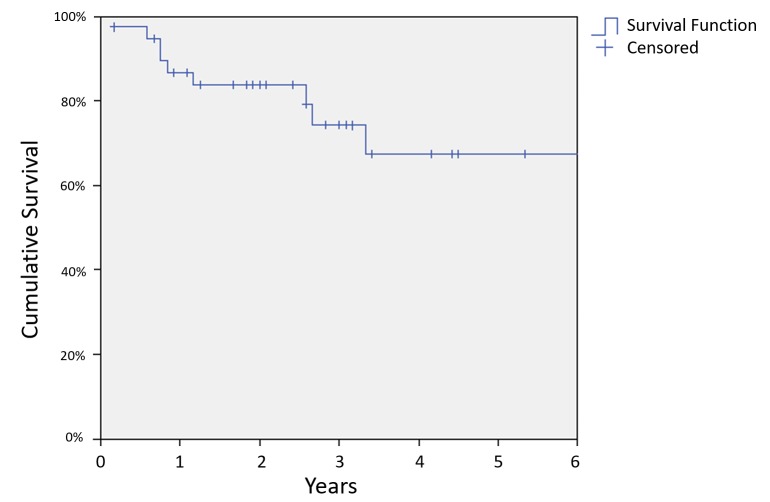
Recurrence-free survival

Median follow-up was 2.6 years with a range of 0.2 years to 10 years. Seven patients died during follow-up, and the three-year overall survival (OS) was 83% (Figure 1). There were six deaths due to NPC, and the three-year DSS was 86% (Figure 2). Nine patients had documented local or regional recurrences following treatment with a three-year LRRFS of 74% (Figure 3).

## Discussion

This retrospective study of locally advanced NPC patients evaluated the impact of neoadjuvant cisplatin on concurrent cisplatin chemotherapy dose. Higher doses of neoadjuvant chemotherapy were associated with completing more cycles of concurrent cisplatin during the radiotherapy stage of treatment. This was in opposition to our hypothesis that increasing the overall cisplatin exposure and the associated toxicity would decrease the amount of concurrent cisplatin received during radiation.

Neoadjuvant regimens for NPC are typically platinum-based combinations. The goal of therapy includes reducing the primary tumor and controlling the microscopic metastatic disease. Multiple Phase II trials have been conducted using cisplatin in combination with anthracyclines, taxanes, and anti-metabolites [7-8,10-11]. The total dose of neoadjuvant cisplatin in these studies ranged from 150 mg/m^2^ to 240 mg/m^2^ with a concurrent component total dose of 150 mg/m^2^ to 300 mg/m^2^. Despite these cumulatively high doses of cisplatin, compliance with therapy was reported to be high in these patients selected for the clinical trial.

Nephrotoxicity, neuropathy, and ototoxicity have been recognized as treatment-limiting side effects with cisplatin. A cumulative dose of cisplatin of over 400 mg/m^2^ via bolus administration or concurrent delivery with radiotherapy is known to increase the likelihood of developing these side effects. Established predictive patient factors include increased age and female sex. In our study, the dose of concurrent cisplatin was not negatively impacted using neoadjuvant treatment despite the anticipated higher risk of toxicity. Like other studies, however, male sex appears to be predictive of the ability to receive higher doses of concurrent cisplatin.

It is difficult to compare survival outcomes from different studies due to the subgroup of bulky tumors that were selected for this treatment at our center, but the three-year OS rate of 83% for locally advanced NPC is comparable to several Phase II clinical trials of neoadjuvant chemotherapy prior to radical treatment for NPC [7-8]. Neoadjuvant chemotherapy for NPC has been evaluated prospectively among few patients. Hui et al. reported on a Phase II trial of 65 patients randomized to neoadjuvant treatment for Stage III and IV-B NPC followed by chemoradiation in Hong Kong. The three-year progression-free survival (PFS) reported for neoadjuvant versus control was 88.2% and 59.5%, and the three-year OS for neoadjuvant versus control was 94.1% and 67.7%, respectively [8]. Kong et al. in Shanghai reported on three-year PFS and OS for 52 patients with Stage III NPC and 64 patients with non-metastatic Stage IV NPC all treated with neoadjuvant chemotherapy followed by concurrent chemoradiation. They report a three-year PFS at 78.2% and 85.1% for Stage III and IV patients, respectively. The three-year OS in this trial was 94.8% and 90.2% for Stage III and IV patients, respectively [7]. However, in these two trials, a bulky primary tumor was not part of the inclusion criteria. Nonetheless, these results are comparable to our results. A previous retrospective analysis by Hamilton et al. of NPC patients at the BCCA reported five-year survival outcomes for Stage III and IVB patients at close to our three-year survival results [12].

Limitations of this study design include the fact that it was a retrospective study and all toxicities of neoadjuvant therapy may not have been captured. There could be a selection bias in treatment decisions, both in selecting patients for neoadjuvant therapy who were more fit at diagnosis and patients with a high tumor stage for cytoreduction. The neoadjuvant and concurrent chemotherapy schedules and regimens were not uniform; however, we were able to collect the total cisplatin doses in each phase. The total dose of cisplatin > 200 mg/m^2^ as a marker for tolerating concurrent cisplatin chemotherapy was chosen based on existing literature and may not be the best marker for clinical effectiveness. Despite these design limitations, this is a fairly large cohort of locally advanced NPC patients. So far, studies of neoadjuvant chemotherapy before concurrent chemoradiation have been limited, particularly in the North American setting. There are currently ongoing Phase III randomized controlled trials (Hong Kong, Singapore, and France) addressing neoadjuvant chemotherapy prior to concurrent chemoradiotherapy for NPC. Sun et al. recently published a Phase III trial of neoadjuvant chemotherapy for locally advanced nasopharyngeal carcinoma. They report significantly improved failure-free survival at three years for the neoadjuvant chemotherapy arm [13].

Gross tumor volume downsizing for the radiation treatment is one of the intended goals of neoadjuvant chemotherapy. In this study, we did not quantify the effect of neoadjuvant chemotherapy on tumor shrinkage. However, the magnitude of dosimetric optimization after neoadjuvant chemotherapy is a topic of interest. A study of the volumetric effects of neoadjuvant chemotherapy is underway at our institution.

## Conclusions

Locally advanced NPC patients have been treated with neoadjuvant chemotherapy at this center with favorable outcomes. Most patients could tolerate both neoadjuvant cisplatin-based chemotherapy and concurrent cisplatin chemotherapy. Higher doses of neoadjuvant chemotherapy and male sex were associated with receiving > 200 mg/m^2^ concurrent cisplatin. It appears that neoadjuvant cisplatin chemotherapy is tolerable prior to concurrent cisplatin with radiotherapy and increasing neoadjuvant cisplatin exposure is not a limiting factor for concurrent chemotherapy delivery.
